# Angiopoietin-like 4 promotes melanoma cell invasion and survival through aldolase A

**DOI:** 10.3892/ol.2014.2071

**Published:** 2014-04-16

**Authors:** YANG SUN, JIANHONG LONG, YU ZHOU

**Affiliations:** 1 Department of Plastic and Aesthetic Surgery, Xiangya Hospital, Central South University, Changsha, Hunan 410008, P.R. China; 2 Department of Orthopaedics, The Second Xiangya Hospital, Central South University, Changsha, Hunan 410008, P.R. China

**Keywords:** angiopoietin-like 4, aldolase A, melanoma, cell invasion, cell survival, protein kinase C, matrix metalloproteinase-2

## Abstract

In the present study, the association between angiopoietin-like 4 (ANGPTL4) and aldolase A (ALDOA) in human melanoma cell invasion and survival was investigated. Overexpression and knockdown of ANGPTL4 were respectively performed in WM-115 and WM-266-4 cells. ALDOA expression at both the mRNA and the protein levels as well as the *ALDOA* gene promoter activities were increased and decreased in parallel with overexpression and knockdown of ANGPTL4 in the melanoma cells, which was blocked by selective protein kinase C (PKC) inhibitor and restored by PKC agonist, respectively. ANGPTL4 overexpression significantly increased cell invasion and matrix metalloproteinase-2 (MMP-2) expression and decreased cell apoptosis against cisplatin in WM-115 cells, which was reversed by knocking down ALDOA. In WM-266-4 cells, knockdown of ANGPTL4 decreased cell invasion and MMP-2 expression and increased cell apoptosis against cisplatin, which was reversed by overexpression of ALDOA. In conclusion, this study demonstrates that ANGPTL4 upregulates ALDOA expression in human melanoma cells at the ALDOA gene promoter/transcriptional level through a PKC-dependent mechanism, and that ALDOA is a critical mediator of the promoting effect of ANGPTL4 on melanoma cell invasion, likely through upregulating the MMP-2 expression. Additionally, our results suggest that ALDOA plays an important role in ANGPTL4-enhanced melanoma cell survival against apoptotic stress, which implicates ANGPTL4 and ALDOA in the development of melanoma chemoresistance.

## Introduction

A novel approach to therapeutic strategy is emerging, which based on the peculiar metabolism of cancer cells. Cancer cells are characterized by a high rate of glycolysis, which is their primary energy source, exceeding the capacity of mitochondrial oxidative energy metabolism (1). Fructose-bisphosphate aldolase (EC 4.1.2.13) is involved in glycolysis by converting fructose 1,6-diphosphate into dihydroxyacetone phosphate and glyceraldehyde-3-phosphate (2). The three aldolase isozymes (ALDOA, ALDOB and ALDOC) have a tetramer structure with identical molecular weights of ~160 kDa. It is well known that cancer cells with a high glycolytic rate often exhibit an aberrant expression of all glycolytic enzymes (2). It has been found that the control of glycolysis in rapidly growing tumor cells occurs at least partly at the level of the consuming block (from aldolase to lactate dehydrogenase) (3). Accumulation of fructose-1,6-bisphosphate resulting from inhibition of aldolase-catalyzed cleavage should stop glycolysis and, therefore, cancer development and progression (2). A previous study has suggested that aldolase is involved in melanoma cell survival (1).

Angiopoietin-like 4 (ANGPTL4), a secreted protein of the angiopoietin-like family, is involved in regulating glucose homeostasis, insulin sensitivity and lipid metabolism through its capacity to inhibit lipoprotein lipase (4–6). A previous study has shown that ANGPTL4 expression is regulated by hypoxia in tumor cells (7). Moreover, ANGPTL4 mRNA is expressed in the perinecrotic areas of various human tumors and is highly upregulated in epithelial tumor cells from clear-cell renal carcinoma (8). A recent study has shown that ANGPTL4 is highly expressed in melanoma brain metastasis and micrometastasis cells, suggesting that ANGPTL4 is involved in melanoma metastasis (9).

To the best of our knowledge, the present study is the first to investigate the relationship between ANGPTL4 and ALDOA in human melanoma cell invasion and survival.

## Materials and methods

### Cell lines, plasmids and reagents

WM-115 and WM-266-4 human melanoma cell lines were purchased from the American Type Culture Collection (Manassas, VA, USA). Human full-length *ANGPTL4* and ALDOA cDNAs (Origene, Beijing, China) were subcloned into pcDNA 3.1 expression vectors (Invitrogen Life Technologies, Carlsbad, CA, USA), respectively (9,10). Human *ALDOA* promoter-luciferase reporter (HPRM14783-PG02) and Secrete-Pair Gaussia Luciferase Assay kit (SPGA-G010) were purchased from GeneCopoeia (Rockville, MD, USA). Human ANGPTL4 (sc-44664-V) and human *ALDOA* (sc-29664-V) shRNA lentiviral particles; control shRNA lentiviral particles-A (sc-108080); and anti-ANGPTL4 (N-15) (sc-34113), -ALDOA (N-15) (sc-12059) and -matrix metalloproteinase-2 (MMP-2) antibodies (sc-53630) were purchased from Santa Cruz Biotechnology, Inc. (Santa Cruz, CA, USA). DeadEnd™ Fluorometric TUNEL system was purchased from Promega (Madison, WI, USA). Superfect™ transfection reagent was purchased from Qiagen (Valencia, CA, USA). Selective protein kinase C (PKC) inhibitor Go6983 and agonist phorbol 12-myristate 13-acetate (PMA), as well as puromycin, G418 and cisplatin were purchased from Sigma-Aldrich (St. Louis, MO, USA).

### Transfection and lentiviral transduction

The ANGPTL4 and ALDOA expression constructs were transfected into cells using Superfect transfection reagent (Qiagen) according to the manufacturer’s instructions. Pools of stable transductants were generated via selection with G418 (800 μg/ml) according to the manufacturer’s protocol. Lentiviral transduction was performed and pools of stable transductants were generated via selection with puromycin (5 μg/ml).

### Western blot analysis

Immunoblotting was performed with respective antibodies. Briefly, cells were dissolved in 250 μl of 2× SDS loading buffer (62.5 mM Tris-HCl, pH 6.8; 2% SDS; 25% glycerol; 0.01% bromphenol blue and 5% 2-mercaptoethanol; Invitrogen Life Technologies), and incubated at 95°C for 10 min. Equal amount of proteins for each sample were separated by 10% SDS-polyacrylamide gel (Invitrogen Life Technologies) electrophoresis and blotted onto a polyvinylidene difluoride microporous membrane (Millipore, Billerica, MA, USA). Membranes were incubated for 1 h with a 1/1000 dilution of anti-ANGPTL4 goat polyclonal (N-15; sc-34113), anti-ALDOA goat polyclonal (N-15; sc-12059) and anti-MMP-2 mouse monoclonal antibodies (sc-53630) (all Santa Cruz Biotechnology, Inc.) and then washed and revealed using mouse anti-goat IgG-B (sc-53799) or donkey anti-mouse IgG-B (sc-2098) secondary antibodies (Santa Cruz Biotechnology, Inc.) with horseradish peroxidase conjugate (1/5000, 1 h). Peroxidase was revealed with an ECL detection plus kit (GE Healthcare, Little Chalfont, UK).

### Quantitative polymerase chain reaction (qPCR)

RNA was prepared from cells using TRIzol reagent (Invitrogen Life Technologies) followed by purification with Turbo DNA-free kit (Ambion, Austin, TX, USA). The cDNAs were synthesized using SuperScript II reverse transcriptase (Invitrogen Life Technologies). Real-time qPCR was performed using an Abi-Prism 7700 sequence detection system (Applied Biosystems, Foster City, CA, USA), using the fluorescent dye SYBR Green Master Mix (PE Biosystems, Framingham, MA, USA) as described by the manufacturer. The results were normalized against that of the housekeeping gene glyceraldehyde-3-phosphate dehydrogenase (*GAPDH*) in the same sample. The primers used are as follows: Forward, 5′-TCATCCTCTTCCATGAGACACTCT-3′ and reverse, 5′-ATTCTGCTGGCAGATACTGGCATAA-3′ for human ALDOA; forward, 5′-GACTCATGACCACAGTCCATGC-3′ and reverse, 5′-AGAGGCAGGGATGATGTTCTG-3′ for human GAPDH. Each experiment was repeated twice and performed in triplicate.

### Luciferase Assay

WM-115 and WM-266-4 cells were transfected with human *ALDOA* promoter-luciferase reporter constructs using Superfect transfection reagent (Qiagen). Plasmid PRL-CMV encoding *Renilla reniformis* luciferase (at one-fifth molar ratio to test plasmids) was co-transfected with test plasmids in each transfection as an internal control for data normalization. Luciferase assays were performed with a Secrete-Pair Gaussia Luciferase Assay kit (GeneCopoeia) according to the manufacturer’s instructions. Each experiment was repeated three times and performed in triplicate.

### In vitro cell invasion assay

Transwell^®^ cell-culture chambers with 8-μm pore size (BD Biosciences, Bedford, MA, USA) for 24-well plates were coated with 50 μl Matrigel (10 mg/ml; BD Biosciences; diluted 1:3 in RPMI-1640; Life Technologies, Grand Island, NY, USA). WM-115 and WM-266-4 cells were seeded in the upper chamber at a density of 5×10^5^ cells per well in RPMI-1640 serum-free medium. Complete medium (600 μl; RPMI-1640 with 5% fetal bovine serum) was added to the lower chamber. Cells were allowed to migrate for 24 h followed by fixation and staining with 1% crystal violet (Sigma-Aldrich). Invaded cells were counted in 10 random fields per chamber under a microscope (BX51-P; Olympus, Guangzhou, China). Each experiment was repeated three times and performed in triplicate.

### Measurement of apoptosis by TUNEL assay

The TUNEL assay was performed using the DeadEnd Fluorometric TUNEL system according to the manufacturer’s instructions (Promega). Cells were treated with cisplatin (10 nM) for 8 h. Apoptotic cells exhibit a strong nuclear green fluorescence that could be detected using a standard fluorescein filter. All cells stained with DAPI exhibit a strong blue nuclear fluorescence. The slides were observed under fluorescence microscopy (AF6000; Leica Microsystems, Beijing, China) with relative apoptotic cells determined by counting TUNEL-positive cells in five random fields (magnification, ×100) per sample.

### Statistical analysis

Statistical analyses were performed with SPSS 10.0 for Windows (SPSS, Inc., Chicago, IL, USA). Data values are expressed as the mean ± standard deviation. Comparisons of means among multiple groups were performed with one-way analysis of variance followed by post hoc pairwise comparisons using Tukey’s tests. A two-tailed P<0.05 was considered to indicate a statistically significant difference.

## Results

### Effect of overexpression and knockdown of ANGPTL4 on ALDOA expression in human melanoma cells

**Figure 1:**
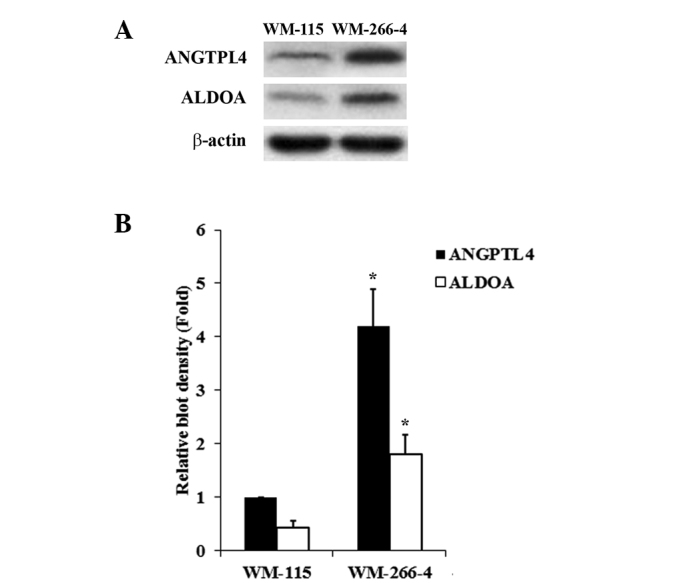
Angiopoietin-like 4 (ANGPTL4) and aldolase A (ALDOA) expression in melanoma cells. (A) ANGPTL4 and ALDOA expression in WM-115 and WM-266-4 human melanoma cells was analyzed with western blot analysis. β-actin blotting was used as a loading control. (B) The density of the ANGPTL4 and ALDOA blots was normalized against that of β-actin to obtain a relative blot density, respectively, which is expressed as the fold-change to the relative ANGPTL4 blot density of WM-115 cells (designated as 1). ^*^P<0.05, compared with WM-115 cells.

**Figure 2:**
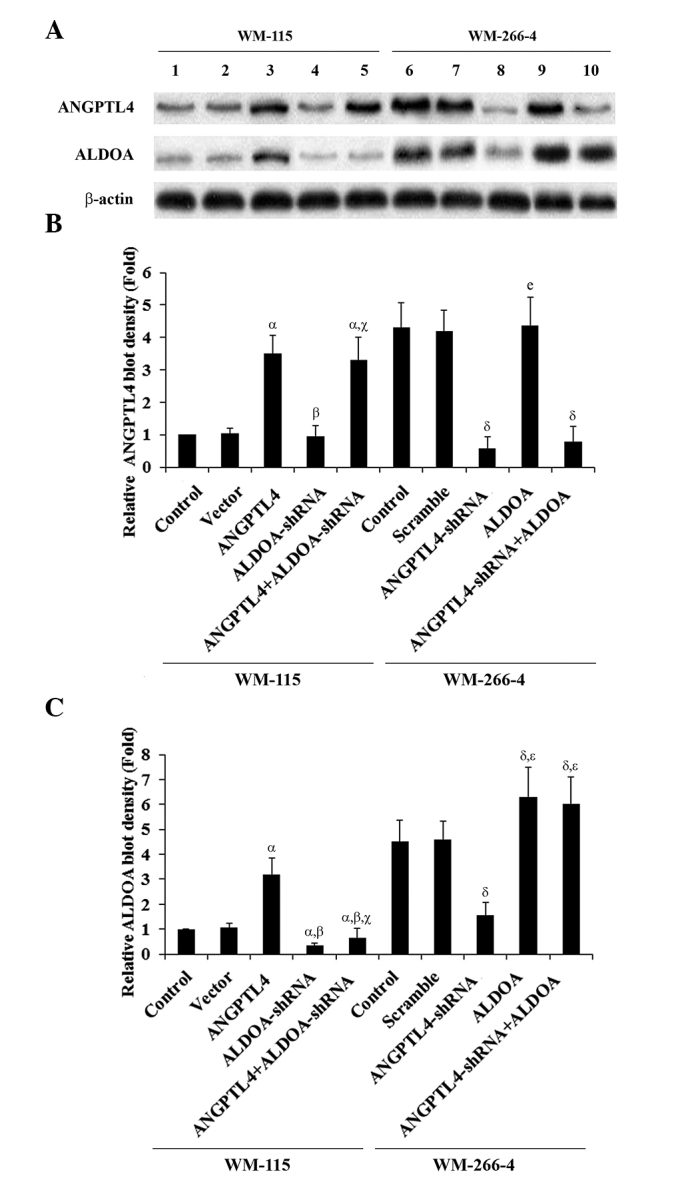
Angiopoietin-like 4 (ANGPTL4) and aldolase A (ALDOA) expression in melanoma cells with overexpression and knockdown of ANGPTL4. (A) WM-115 cells: Expression of ANGPTL4 and ALDOA in control cells (lane 1), cells stably transfected with empty pcDNA3 vector (lane 2), cells overexpressing ANGPTL4 (lane 3), cells treated with selective protein kinase C (PKC) inhibitor Go6983 (500 nM, 24 h; lane 4), and cells overexpressing ANGPTL4 and simultaneously treated with Go6983 (500 nM, 24 h; lane 5), was analyzed by western blotting. WM-266-4 cells: Expression of ANGPTL4 and ALDOA in control cells (lane 6), cells stably transduced with scramble control shRNA (lane 7), cells stably expressing ANGPTL4-shRNA (lane 8), cells treated with selective PKC agonist phorbol 12-myristate 13-acetate (PMA; 500 nM, 24 h; lane 9), and cells stably expressing ANGPTL4-shRNA and simultaneously treated with PMA (500 nM, 24 h; lane 10), was analyzed by western blotting. β-actin blotting was used as a loading control. (B and C) Density of the ANGPTL4 (B) and ALDOA (C) blots was normalized against that of β-actin to obtain a relative blot density, respectively, which was expressed as the fold-change to the relative ANGPTL4 (B) or ALDOA (C) blot density of WM-115 control cells (designated as 1). WM-115 cells: ^α^P<0.05, compared with Control and Vector; ^β^P<0.05, compared with ANGPTL4; ^γ^P<0.05, compared with PKC inhibitor. WM-266-4 cells: ^δ^P<0.05, compared with Control and Scramble; ^χ^P<0.05, compared with ANGPTL4-shRNA.

**Figure 3:**
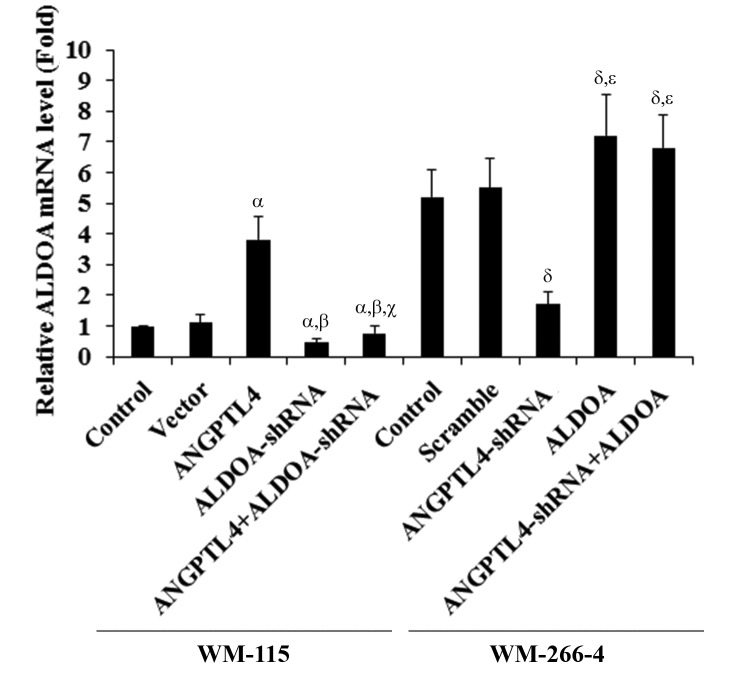
Aldolase A (ALDOA) mRNA expression in melanoma cells with overexpression and knockdown of angiopoietin-like 4 (ANGPTL4). WM-115 cells: The *ALDOA* mRNA level in control cells, cells stably transfected with empty pcDNA3 vector, cells overexpressing ANGPTL4, cells treated with selective protein kinase C (PKC) inhibitor Go6983 (500 nM, 24 h), and cells overexpressing ANGPTL4 and simultaneously treated with Go6983 (500 nM, 24 h), was analyzed by quantitative polymerase chain reaction (qPCR). WM-266-4 cells: The *ALDOA* mRNA level in control cells, cells stably transduced with scramble control shRNA, cells stably expressing ANGPTL4-shRNA, cells treated with selective PKC agonist phorbol 12-myristate 13-acetate (PMA; 500 nM, 24 h, and cells stably expressing ANGPTL4-shRNA and simultaneously treated with PMA (500 nM, 24 h), was analyzed by qPCR. The *ALDOA* mRNA level is shown as the fold-change to that of WM-115 control cells (designated as 1). WM-115 cells: ^α^P<0.05, compared with Control and Vector; ^β^P<0.05, compared with ANGPTL4; ^γ^P<0.05, compared with PKC inhibitor. WM-266-4 cells: ^δ^P<0.05, compared with Control and Scramble; ^χ^P<0.05, compared with ANGPTL4-shRNA.

We employed WM-115 and WM-266-4 human melanoma cells as cellular models in this study. WM-115 was established from a primary melanoma, and WM266-4 was derived from a skin metastatic site of the same tumor from which WM-115 was derived. Western blot analyses showed that WM-115 cells had lower constitutive expression of ANGPTL4 and ALDOA than WM-266-4 cells (Fig. 1). The two cell lines would allow specific ANGPTL4 knockdown or overexpression studies to be performed in the context of the study goals. Thus, we stably transfected WM-115 cells with an ANGPTL4 expression vector to overexpress ANGPTL4, and stably transduced WM-266-4 cells with ANGPTL4-shRNA to knock down ANGPTL4. Western blot analysis showed that stable transfection of ANGPTL4 led to an over two-fold increase of ANGPTL4 expression in WM-115 cells, which was not affected by selective PKC inhibitor Go6983 (500 nM). On the other hand, knockdown of ANGPTL4 by shRNA resulted in a >80% decrease of endogenous ANGPTL4 in WM-266-4 cells, which was not affected by selective PKC agonist PMA (500 nM) (Fig. 2). The ALDOA expression in WM-115 cells increased in parallel with ANGPTL4 overexpression, which was inhibited by Go6983. In WM-266-4 cells, the ALDOA expression decreased in parallel with ANGPTL4 knockdown, which was rescued by PMA (Fig. 2). A similar data trend was observed with *ALDOA* mRNA levels in the cells (Fig. 3).

### Effect of overexpression and knockdown of ANGPTL4 on ALDOA gene promoter activities in human melanoma cells

**Figure 4:**
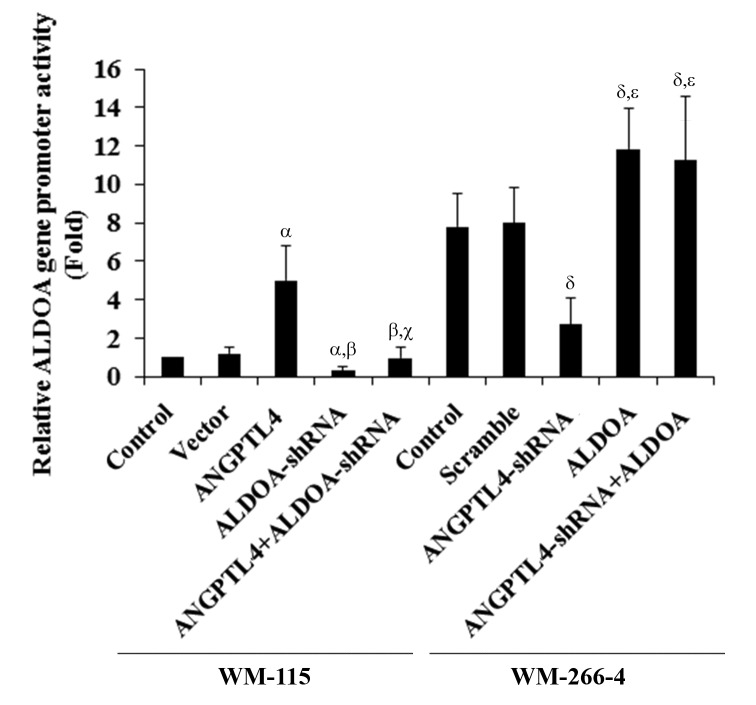
Effect of angiopoietin-like 4 (ANGPTL4) on human aldolase A (ALDOA) promoter activities. WM-115 and WM-266-4 cells were transfected with human *ALDOA* promoter-luciferase reporter plasmids. After 24 h, luciferase assays were performed. WM-115 cells: Luciferase activity in control cells, cells stably transfected with empty pcDNA3 vector, cells overexpressing ANGPTL4, cells treated with selective protein kinase C (PKC) inhibitor Go6983 (500 nM, 24 h), and cells overexpressing ANGPTL4 and simultaneously treated with Go6983 (500 nM, 24 h), was analyzed. WM-266-4 cells: Luciferase activity in control cells, cells stably transduced with scramble control shRNA, cells stably expressing ANGPTL4-shRNA, cells treated with selective PKC agonist phorbol 12-myristate 13-acetate (PMA; 500 nM, 24 h), and cells stably expressing ANGPTL4-shRNA and simultaneously treated with PMA (500 nM, 24 h), was analyzed. The luciferase activity was expressed as the fold-change to that of WM-115 control cells (designated as 1). WM-115 cells: ^α^P<0.05, compared with Control and Vector; ^β^P<0.05, compared with ANGPTL4; ^γ^P<0.05, compared with PKC inhibitor. WM-266-4 cells: ^δ^P<0.05, compared with Control and Scramble; ^χ^P<0.05, compared with ANGPTL4-shRNA.

To determine whether ANGPTL4 regulates ALDOA expression in human melanoma cells by altering the *ALDOA* gene promoter activity, we transfected WM-115 and WM-266-4 cells with human *ALDOA* promoter-luciferase reporter plasmids. Luciferase assays showed that the ALDOA gene promoter activity in WM-115 cells was increased by ANGPTL4 overexpression, which was inhibited by Go6983 (500 nM). In WM-266-4 cells, the *ALDOA* gene promoter activity was decreased by ANGPTL4 knockdown, which was completely restored by PMA (500 nM) (Fig. 4).

### Functional role of ALDOA in ANGPTL4-enhanced cell invasion and MMP-2 expression in human melanoma cells

**Figure 5:**
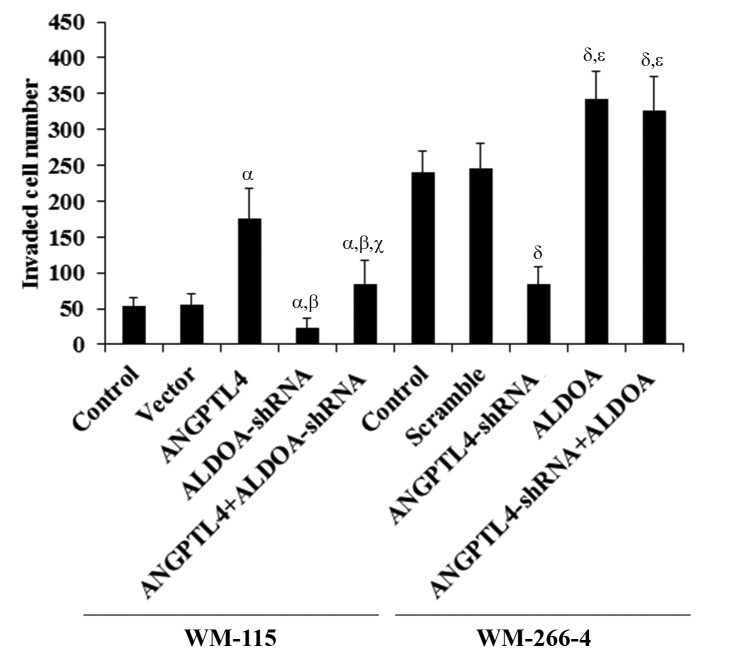
*In vitro* cell invasion in melanoma cells with overexpression and knockdown of angiopoietin-like 4 (ANGPTL4) and/or aldolase A (ALDOA) WM-115 cells: *In vitro* cell invasion assays were performed in control cells, cells stably transfected with empty pcDNA3 vector, cells overexpressing ANGPTL4, cells stably expressing ALDOA-shRNA, and cells overexpressing ANGPTL4 plus stably expressing ALDOA-shRNA. WM-266-4 cells: *In vitro* cell invasion assays were performed in control cells, cells stably transduced with scramble control shRNA, cells stably expressing ANGPTL4-shRNA, cells overexpressing ALDOA, and cells stably expressing ANGPTL4-shRNA plus overexpressing ALDOA. Invaded cell numbers were counted. WM-115 cells: ^α^P<0.05, compared with Control and Vector; ^β^P<0.05, compared with ANGPTL4; ^γ^P<0.05, compared with ALDOA-shRNA. WM-266-4 cells: ^δ^P<0.05, compared with Control and Scramble; ^χ^P<0.05, compared with ANGPTL4-shRNA.

**Figure 6:**
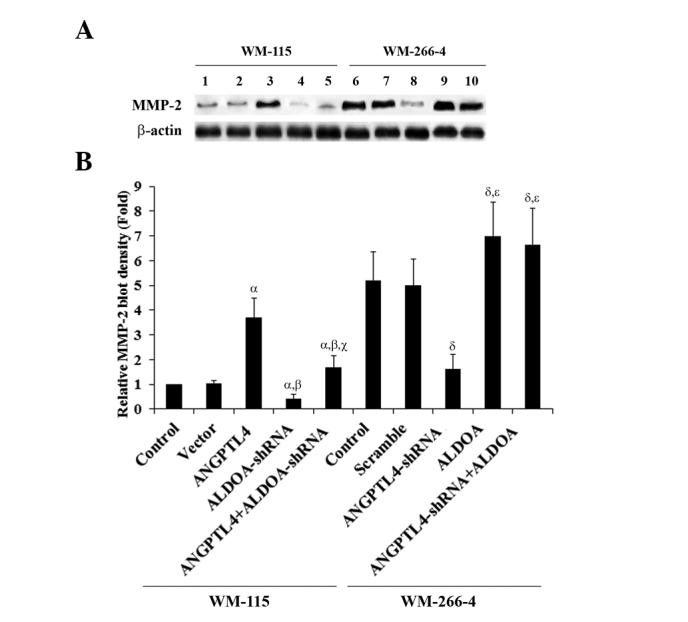
Matrix metalloproteinase-2 (MMP-2) expression in melanoma cells with overexpression and knockdown of angiopoietin-like 4 (ANGPTL4) and/or aldolase A (ALDOA). (A) WM-115 cells: MMP-2 expression was determined in control cells (lane 1), cells stably transfected with empty pcDNA3 vector (lane 2), cells overexpressing ANGPTL4 (lane 3), cells stably expressing ALDOA-shRNA (lane 4), and cells overexpressing ANGPTL4 plus stably expressing ALDOA-shRNA (lane 5), by western blot analysis. WM-266-4 cells: MMP-2 expression was determined in control cells, cells stably transduced with scramble control shRNA (lane 7), cells stably expressing ANGPTL4-shRNA (lane 8), cells overexpressing ALDOA (lane 9), and cells stably expressing ANGPTL4-shRNA plus overexpressing ALDOA (lane 10), by western blot analysis. β-actin blotting was used as a loading control. (B) The density of the MMP-2 blot was normalized against that of β-actin to obtain a relative blot density, which was expressed as the fold-change to the relative MMP-2 blot density of WM-115 control cells (designated as 1). WM-115 cells: ^α^P<0.05, compared with Control and Vector; ^β^P<0.05, compared with ANGPTL4; ^γ^P<0.05, compared with ALDOA-shRNA. WM-266-4 cells: ^δ^P<0.05, compared with Control and Scramble; ^χ^P<0.05, compared with ANGPTL4-shRNA.

To examine the functional roles of ANGPTL4 and ALDOA in melanoma cell invasion, we performed *in vitro* cell invasion assays, which showed that ANGPTL4 overexpression increased cell invasion in WM-115 cells by over two-fold, which was reversed by knocking down ALDOA (Fig. 5). In WM-266-4 cells, knockdown of ANGPTL4 decreased cell invasion by over 65%, which was completely restored by overexpression of ALDOA (Fig. 5). A similar data trend was observed with MMP-2 expression in the cells (Fig. 6).

### Functional role of ALDOA in ANGPTL4-enhanced cell survival against cisplatin in human melanoma cells

**Figure 7:**
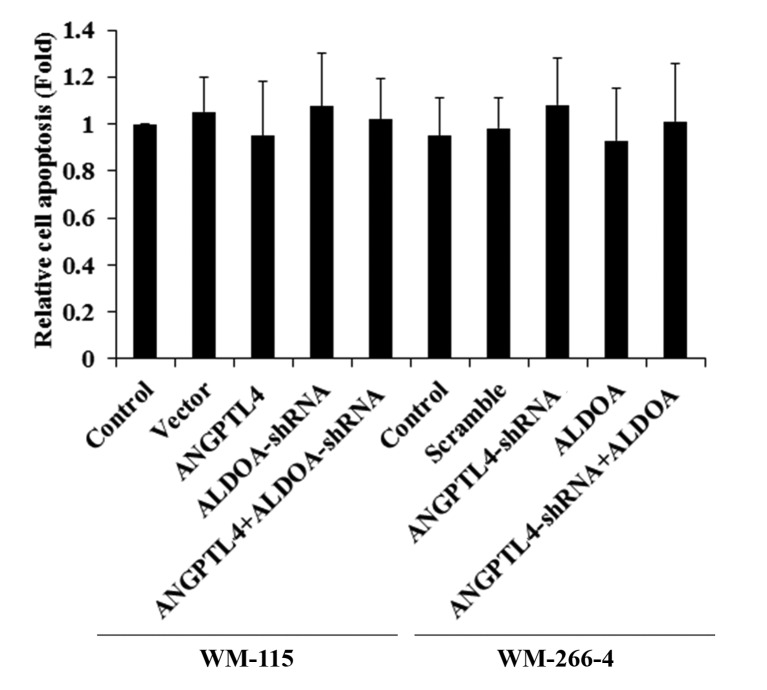
Apoptosis in melanoma cells with overexpression or knockdown of angiopoietin-like 4 (ANGPTL4) and/or aldolase A (ALDOA) under normal culture conditions. WM-115 cells: TUNEL assays were performed in control cells, cells stably transfected with empty pcDNA3 vector, cells overexpressing ANGPTL4, cells stably expressing ALDOA-shRNA, and cells overexpressing ANGPTL4 plus stably expressing ALDOA-shRNA. WM-266-4 cells: TUNEL assays were performed in control cells, cells stably transduced with scramble control shRNA, cells stably expressing ANGPTL4-shRNA, cells overexpressing ALDOA, and cells stably expressing ANGPTL4-shRNA plus overexpressing ALDOA. The cells were maintained under normal culture conditions for 8 h. The cell apoptosis rate at 8 h was expressed as the fold-change to that of WM-115 control cells (designated as 1).

**Figure 8:**
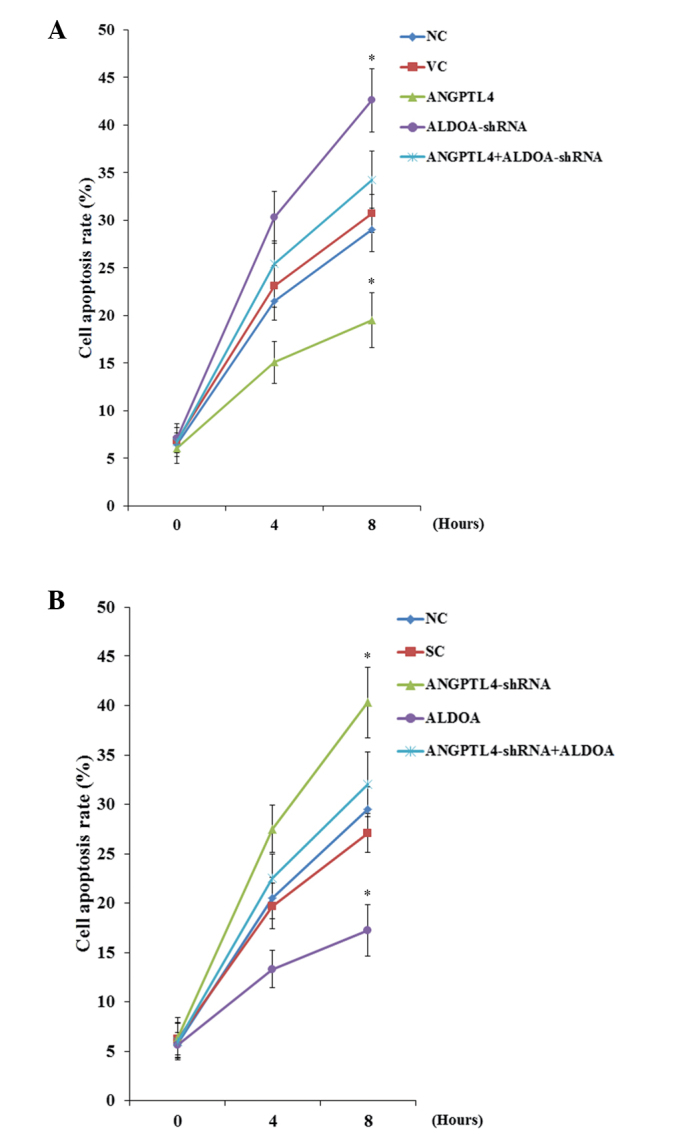
Cisplatin-induced apoptosis in melanoma cells with overexpression or knockdown of angiopoietin-like 4 (ANGPTL4) and/or aldolase A (ALDOA). (A) WM-115 cells: TUNEL assays were performed in control cells, cells stably transfected with empty pcDNA3 vector, cells overexpressing ANGPTL4, cells stably expressing ALDOA-shRNA, and cells overexpressing ANGPTL4 plus stably expressing ALDOA-shRNA. (B) WM-266-4 cells: TUNEL assays were performed in control cells, cells stably transduced with scramble control shRNA, cells stably expressing ANGPTL4-shRNA, cells overexpressing ALDOA, and cells stably expressing ANGPTL4-shRNA plus overexpressing ALDOA. The cells were treated with 10 nM of cisplatin for 8 h. Cell apoptosis rates at 4 and 8 h were shown as the percentage of TUNEL positive cells in total cells. WM-115 cells: ^*^P<0.05, compared with Control and Vector. WM-266-4 cells: ^*^P<0.05, compared with Control and Scramble.

To investigate the functional roles of ANGPTL4 and ALDOA in melanoma cell survival against apoptotic stress, we examined cell apoptosis in melanoma cells treated with 10 nM of cisplatin, an apoptosis-inducing chemotherapeutic agent commonly used to treat melanoma. Overexpression or knockdown of ANGPTL4 and/or ALDOA did not significantly alter cell apoptosis in both WM-115 and WM-266-4 cells under normal culture conditions (Fig. 7). However, in WM-115 cells treated with cisplatin, overexpression of ANGPTL4 significantly decreased cell apoptosis compared with the controls, which was reversed by knocking down ALDOA (Fig. 8A). In WM-266-4 cells, knockdown of ANGPTL4 significantly increased cisplatin-induced cell apoptosis, which was reversed by overexpression of ALDOA (Fig. 8B).

## Discussion

Inhibiting cancer cell glycolysis is an emerging therapeutic strategy for cancer (2). A previous study suggested that aldolase is involved in melanoma cell survival (1). ANGPTL4 reportedly is involved in melanoma metastasis (9). To the best of our knowledge, the present study provides the first evidence that ANGPTL4 upregulates ALDOA expression in human melanoma cells, and that a major part of the promoting effect of ANGPTL4 on melanoma cell invasion and survival is mediated by ALDOA.

WM-115 and WM-266-4 cells were utilized as melanoma cell models in this study. The two cell lines were respectively established from a primary melanoma and a skin metastatic site of the same tumor in the same patient, which gives them a more comparable genetic background. In addition, WM-115 cells express a relatively low level of ANGPTL4 compared with WM-266-4 cells. Thus, overexpression and knockdown of ANPTL-4 were respectively performed in the two cell lines to approach the study objectives from different angles.

ANGPTL4 reportedly modulates epidermal differentiation through stimulating the expression of PKC (11), and stimulation of PKC has been shown to promote *ALDOA* gene transcription (12). In the present study, ALDOA expression at both the mRNA and the protein levels was significantly increased and decreased in parallel with overexpression and knockdown of ANGPTL4 in melanoma cells, which was blocked by selective PKC inhibitor and restored by PKC agonist, respectively. The results suggest that ANGPTL4 expression may affect ALDOA expression in human melanoma cells at the gene transcription level through a PKC-dependent mechanism. Luciferase assays confirmed that ANGPTL4 could enhance *ALDOA* gene promoter/transcriptional activities in melanoma cells through a PKC-dependent mechanism. However, the mechanism by which ANGPTL4 modulates the *ALDOA* promoter activities remains unclear and will be further investigated in our future studies. In addition, although it has been reported that activation of PKC can induce ANGPTL4 expression in human airway smooth muscle cells (13), our data indicate that PKC does not modulate ANTPTL-4 expression in melanoma cells.

A previous study suggested that ANGPTL4 may promote melanoma metastasis (9). Since our findings had suggested that ALDOA was a downstream effector of ANGPTL4/PKC signaling, we investigated the functional roles of ANGPTL4 and ALDOA in melanoma cell invasion. ALDOA knockdown almost canceled the effects of increased cell invasion and MMP-2 expression caused by ANGPTL4 overexpression in WM-115 cells, while ALDOA overexpression restored the decreased cell invasion and MMP-2 expression caused by ANGPTL4 knockdown in WM-266-4 cells. The results suggest that ALDOA is a critical mediator of the promoting effect of ANGPTL4 on melanoma cell invasion, likely through upregulating the MMP-2 expression.

Cell survival against apoptotic stress is critical for cancer progression and metastasis (14). In the current study, a relatively small concentration of cisplatin (10 nM) was used to induce apoptotic stress without killing the majority of the cells. In the presence of cisplatin, ALDOA knockdown almost canceled the effects of increased cell survival caused by ANGPTL4 overexpression in WM-115 cells, while ALDOA overexpression restored the decreased cell survival caused by ANGPTL4 knockdown in WM-266-4 cells. The results not only suggest an important functional role of ALDOA in ANGPTL4-enhanced melanoma cell survival, but also implicate ANGPTL4 and ALDOA in the development of melanoma chemoresistance. Cisplatin elicits DNA repair mechanisms by crosslinking DNA, which in turn activates apoptosis when repair proves impossible (14). It remains unclear whether ANGPTL4 and ALDOA may impact melanoma cell survival against other types of chemotherapy agents. Further studies with additional types of chemotherapy agents and melanoma cell lines would elaborate this issue.

The aldolase isozymes (ALDOA, ALDOB and ALDOC) are encoded by three different genes, differentially expressed during development. ALDOA is mainly produced by the developing embryo and in adult muscle; ALDOB is produced by the liver, kidney and intestine; and ALDOC is mainly produced by the brain and other nervous tissue. ALDOA and ALDOB have been associated with poor prognosis of osteosarcoma and hepatocarcinoma, respectively (15,16). It would be interesting to explore in future studies whether and how ALDOB and ALDOC are involved in melanoma cell invasion and survival.

In conclusion, the present study demonstrates that ANGPTL4 upregulates ALDOA expression in human melanoma cells at the ALDOA gene promoter/transcriptional level through a PKC-dependent mechanism, and that ALDOA is a critical mediator of the promoting effect of ANGPTL4 on melanoma cell invasion, likely through upregulating the MMP-2 expression. Additionally, our results also suggest that ALDOA plays an important role in ANGPTL4-enhanced melanoma cell survival against cisplatin-induced apoptotic stress, which implicates ANGPTL4 and ALDOA in the development of melanoma chemoresistance.
